# Alpha-diversity and microbial community structure of the male urinary microbiota depend on urine sampling method

**DOI:** 10.1038/s41598-021-03292-x

**Published:** 2021-12-09

**Authors:** Jan Hrbacek, Daniel Morais, Pavel Cermak, Vitezslav Hanacek, Roman Zachoval

**Affiliations:** 1 Department of Urology, 3rd Faculty of Medicine, Charles University, Thomayer University Hospital, Videnska 800, 14059 Prague, Czech Republic; 2 Laboratory of Environmental Microbiology, Institute of Microbiology, Czech Academy of Sciences, Videnska 1083, 14200 Prague, Czech Republic; 3 Department of Clinical Microbiology, Thomayer University Hospital, Videnska 800, 14059 Prague, Czech Republic

**Keywords:** Microbiome, Bladder, Prostate, Urethra

## Abstract

Considerable variation exists in the methodology of urinary microbiota studies published so far including the cornerstone of any biomedical analysis: sample collection. The aim of this study was to compare the urinary microbiota of first-catch voided urine (FCU), mid-stream voided urine (MSU) and aseptically catheterised urine in men and define the most suitable urine sampling method. Forty-nine men (mean age 71.3 years) undergoing endoscopic urological procedures were enrolled in the study. Each of them contributed three samples: first-catch urine (FCU), mid-stream urine (MSU) and a catheterised urine sample. The samples were subjected to next-generation sequencing (NGS, n = 35) and expanded quantitative urine culture (EQUC, n = 31). Using NGS, *Bacteroidetes*, *Firmicutes,* and *Proteobacteria* were the most abundant phyla in our population. The most abundant genera (in order of relative abundance) included: *Prevotella*, *Veillonella*, *Streptococcus*, *Porphyromonas*, *Campylobacter*, *Pseudomonas*, *Staphylococcus*, *Ezakiella*, *Escherichia* and *Dialister*. Eighty-two of 105 samples were dominated by a single genus. FCU, MSU and catheterised urine samples differed significantly in three of five alpha-diversity measures (ANOVA, *p* < 0.05): estimated number of operational taxonomic units, Chao1 and abundance-based coverage estimators. Beta-diversity comparisons using the PIME method (Prevalence Interval for Microbiome Evaluation) resulted in clustering of urine samples according to the mode of sampling. EQUC detected cultivable bacteria in 30/31 (97%) FCU and 27/31 (87%) MSU samples. Only 4/31 (13%) of catheterised urine samples showed bacterial growth. Urine samples obtained by transurethral catheterisation under aseptic conditions seem to differ from spontaneously voided urine samples. Whether the added value of a more exact reflection of the bladder microbiota free from urethral contamination outweighs the invasiveness of urethral catheterisation remains to be determined.

## Introduction

The human urinary tract had traditionally been considered a sterile environment unlike other body niches such as the gut, oropharynx or vagina; hence, it was not included in the Human Microbiome Project^1^. However, the last decade has brought evidence of microbial communities residing in the female^2,3^ and male^4,5^ urinary tract. Various microorganisms have been cultured from urine using expanded quantitative urinary culture (EQUC)^6^ and much more prokaryotic diversity unearthed using next-generation sequencing (NGS)^7,8^.

Female urinary microbiota has been given a broad attention; it was less so in case of male urinary microbiota (MUM). And while the microbiota of the female urinary bladder overlaps with that of the vagina to the point that a term “female urogenital microbiota” has been suggested^9^, little is known about potential differences in the microbiota along the male urinary tract. According to a study from 2011, *Lactobacillus, Sneathia, Veillonella, Corynebacterium* and *Prevotella* were the abundant bacterial genera detected in male first-catch urine (FCU) as well as urethral swab specimens^4^. Anecdotic evidence has suggested certain differences between microbial compositions of voided and catheterised urine samples in males^7,10,11^. Differences in beta- (but not alpha-) diversity were reported in a study of urinary bladder cancer patients between voided and cystoscopy-obtained urine samples^10^. Another study reported that urethra and bladder microbiomes did not differ in their taxonomic composition but rather in taxonomic structure (relative abundances of several genera)^11^. And while the prevalence of bacteria in catheterised urine correlated with the degree of lower urinary tract symptoms in men with enlarged prostates, this relationship was not demonstrated on voided samples^7^.

Alterations of the urinary microbiome have been associated with functional^12–14^ and anatomical^7^ abnormalities of the urinary tract and with the presence of genitourinary malignant disease^15,16^. Urge urinary incontinence was associated with higher microbiota diversity^12,13^ and increased diversity also correlated with poor response to anticholinergic (for bladder overactivity) treatment^12^. Changes in the urinary microbiota were reported in patients after spinal cord injury^14^ and even among patients with chronic kidney disease where lower diversity was associated with more advanced renal insufficiency^13^. Patients with bladder cancer were reported to have higher microbiota richness compared to healthy controls^15^. Chronic pelvic pain syndrome was associated with a greater alpha-diversity and greater prevalence of anaerobic bacteria than urine of controls without the condition^17^. Conversely, women with intersticial cystitis/painful bladder syndrome had less diverse microbiota than controls^19^.

Despite studies looking for and finding differences in composition of the urinary microbiota in a variety of clinical scenarios, an elementary methodological question—i.e. how to collect urine samples to ensure an exact reflection of the urinary bladder microbiota and comparability of future studies—remains unresolved.

The aim of the present study was to describe male urinary microbiota of first-catch voided urine (FCU), mid-stream voided urine (MSU) and aseptically catheterised urine in subjects without symptoms of a urinary tract infection (UTI) and with a negative standard urine culture result. Secondly, we aimed to identify the most suitable sample collection method for the characterization of bacterial communities residing in the male urinary tract.

## Results

### Study population

A total of 49 men (147 urine samples) were included in the study between August 2019 and February 2021 in whom all three urine samples and full clinical information were available. EQUC and NGS were performed on urine samples from 31 and 35 subjects, respectively. Median age of participants was 71.3 years (IQR 63–76). Thirty-one men were undergoing endoscopic urological procedures for urinary bladder cancer while 18 had a benign condition (benign prostate hyperplasia [n = 9] or upper urinary tract stone disease [n = 9]). Standard urine culture with a negative result was performed as part of preoperative assessment 9 days (median; IQR 4–14) before procedure.

Details on clinical and demographic data of the study population are provided in Table 1.

**Table 1:** Clinical and demographic variables of the study population (n = 49).

Variable	
Age, mean (IQR)	71.3 (63–76)
Ethnicity: Caucasian, n (%)	49 (100%)
BMI, mean (IQR)	28.7 (26–32)
Hypertension, n (%)	37 (76%)
Diabetes, n (%)	17 (35%)
Hyperlipidemia, n (%)	23 (47%)
Chronic kidney disease^1^, n (%)	10 (20%)
Bladder cancer, n (%)	31 (63%)
History of instillation treatment for bladder cancer^2^, n (%)	4 (8%)
History of pelvic radiotherapy, n (%)	3 (6%)
Current smoker, n (%)	11 (22%)
Post-void residual urine^3^, n (%)	8 (16%)

*IQR* interquartile range, *BMI* body mass index.

^1^Estimated glomerular filtration rate (CKD-EPI eGFR) < 60 mL/min.

^2^Mitomycin-C, epirubicin or Bacillus Calmette-Guérin.

^3^40 mL or more left in the bladder after spontaneous voiding as measured by ultrasound.

### NGS shows differences in alpha and beta diversity according to sample collection method

Triplets of urine samples from 38 men were subjected to NGS. Three were excluded, as some of their samples did not provide a sufficient amount of DNA sequences leaving 35 subjects for analysis.

A total of 2,208,522 raw reads were obtained from 105 urine samples with a median number of 16,674 reads per sample. After filtering out low quality sequences, removing chimeras and non bacterial DNA we were left with 732,779 reads and a median number of 3166 reads per sample. After exclusion of singletons and doubletons (sequences detected once or twice in the whole dataset), the reads were assigned to 2645 OTUs, 53 phyla, 107 classes, 207 orders, 400 families and 995 genera.

**Figure 1:**
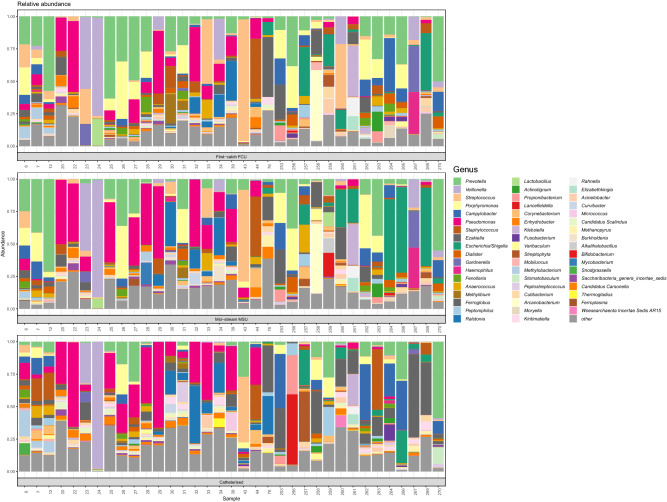
Barplots representing the relative abundance of OTUs in first-catch, mid-stream and catheterised urine samples, respectively, at genus level.

The phyla *Bacteroidetes, Firmicutes,* and *Proteobacteria* represented the majority of sequences in the three types of urine specimens with differing relative abundances. The 10 most abundant genera (in order of relative abundance) were: *Prevotella*, *Veillonella*, *Streptococcus*, *Porphyromonas*, *Campylobacter*, *Pseudomonas*, *Staphylococcus*, *Ezakiella*, *Escherichia* and *Dialister* representing around 530,000 sequences, approximately 72% of the total sequences obtained after removing errors and contaminants. Figure 1 depicts the relative abundances at genus level in all samples.

**Figure 2:**
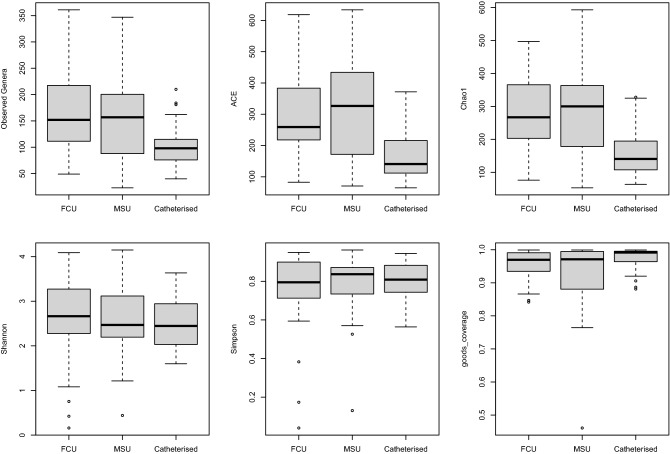
Boxplots comparing alpha-diversity measures and Good´s coverage for first-catch (FCU), mid-stream (MSU) and catheterised urine. See also Table 2. Thick line, median; box limits, upper and lower quartiles; whiskers, 1.5 × interquartile range; points, outliers.

FCU, MSU and catheterised urine samples differed significantly using ANOVA in three out of five alpha diversity measures used: estimated number of OTUs (*p* = 0.0002), Chao1 (*p* < 0.0001) and abundance-based coverage estimators (ACE) index (*p* < 0.0001); there were no statistically significant differences in Simpson and Shannon indices (Table 2; Fig. 2). Good´s coverage values were 0.96, 0.92 and 0.97 for FCU, MSU and catheterised urine, respectively (*p* = 0.02) (Fig. 2). No significant differences in the counts of raw or quality filtered reads were detected.

**Table 2:** Alpha diversity measures (mean values) for first-catch, mid-stream and catheterised urine.

	FCU	MSU	Catheterised	*p* (ANOVA)
Observed genera	171.3	157.8	103.9	0.0002
Chao1	285.1	280.0	160.6	< 0.0001
ACE	315.6	307.3	168.3	< 0.0001
Shannon	2.62	2.61	2.49	0.66
Simpson	0.76	0.79	0.80	0.50

*FCU* first-catch urine, *MSU* mid-stream urine.

**Figure 3:**
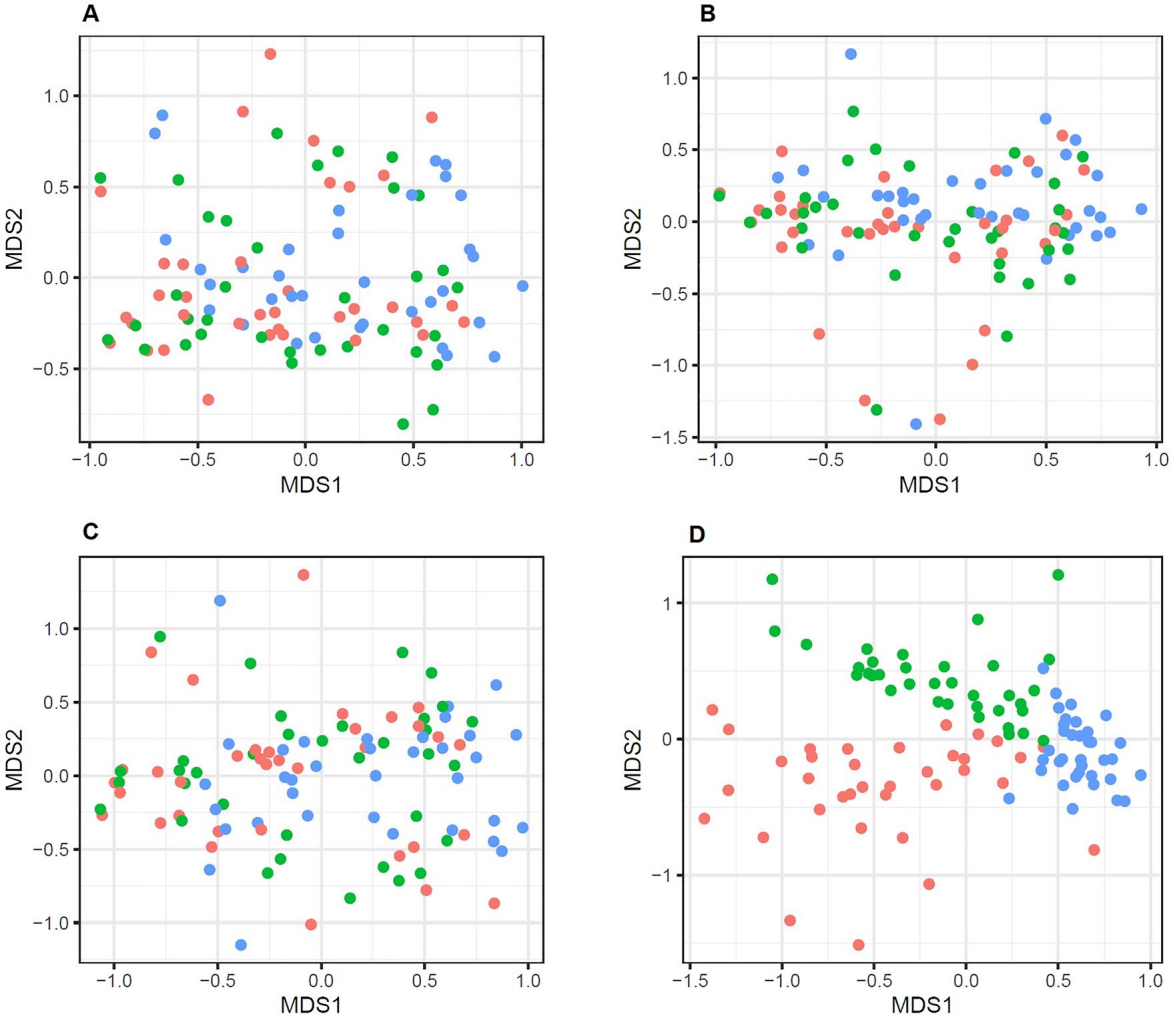
Bacterial community structures of urine depending on the way of sampling (red, first-catch urine; green, mid-stream urine; blue, catheterised urine), under 4 different filtering of OTUs (**A**–**D**). NMDS ordination plots showing the dispersion of samples by their dissimilarity (Bray–Curtis index) after removal of erroneus sequences: PERMANOVA *p* = 0.02, R^2^ = 0.027 (**A**), after keeping only the top 3 OTUs per sample: PERMANOVA *p* = 0.01, R^2^ = 0.039 (**B**), after keeping the 100 most abundant OTUs in the whole dataset: PERMANOVA *p* = 0.02, R^2^ = 0.037 (**C**) and after filtering the most descriptive OTUs based on their prevalence using the PIME algorithm: PERMANOVA *p* = 0.01, R^2^ = 0.348 (**D**).

**Figure 4:**
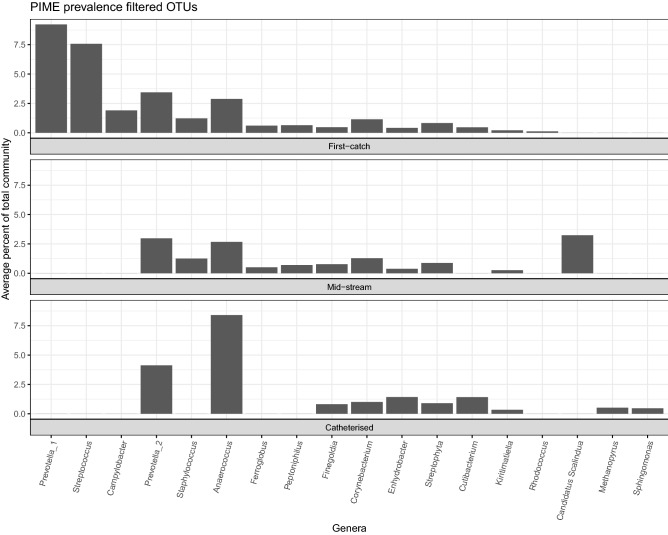
Relative abundance of 18 meaningful OTUs selected by PIME to discriminate among different urine sampling approaches. See also Supplementary Tables S3 and S4. Note: Prevotella_1 and Prevotella_2 correspond to CL0001 and CL0008, respectively, in the Supplementary Table S3.

Beta-diversity comparisons using the PIME method (removing noisy OTUs from samples and keeping only the OTUs that are adding biological meaning to the group of samples) resulted in clear clustering of urine samples according to the mode of sampling (PERMANOVA, *p* < 0.05, Fig. 3). Figure 4 compares the relative abundances by sampling method of 18 OTUs selected by PIME.

Regardless of whether urine samples originated from FCU, MSU or catheterisation, they were mostly dominated (relative abundance > 0.57) by a single genus (82/105 samples, 78.1%), less commonly two (21/105, 20.0%) or three genera (2/105, 1.9%) were evenly represented (the second most abundant OTU´s relative abundance was > 75% of the most abundant one). *Prevotella, Porphyromonas* and *Pseudomonas* were the most frequently found genera among the top-three in the FCU and MSU samples while *Pseudomonas, Ferroglobus* (see the relevant part of the Discussion) and *Prevotella* featured most often amont the top-three in catheterised urine specimens.

### EQUC fails to detect living bacteria in catheterised urine

Among 31 FCU samples subjected to EQUC, 30 (97%) showed presence of cultivable bacteria. MSU specimens yielded a positive EQUC result in 27 (87%) cases. Only four of 31 (13%) specimens obtained via transurethral instrumentation were EQUC-positive.

A total of 23 individual species and 13 genera were identified using EQUC with a median of 2 (range 1–4) isolates per urine sample (of those with a positive result). *Enterococcus faecalis* and *Staphylococcus epidermidis* were the most prevalent (10 of 31 each, 32%) among both FCU and MSU samples, respectively. *Corynebacterium glucuronolyticum* was the third most frequent microorganism isolated (8/31 [26%] and 7/31 [23%] in FCU and MSU, respectively). Among specimens drawn under aseptic conditions from the urinary bladder during theatre procedures, only three yielded a positive EQUC result: *C. glucuronolyticum* in one, *E. faecalis* in two and *S. epidermidis* in one patient. All of these four microorganisms were also present in the particular subject´s FCU and MSU samples.

In terms of concordance between FCU and MSU samples, both showed identical EQUC species-level result in 21 cases (68%). In the remaining 10 patients, the results were discordant as follows: a positive first-catch with a sterile mid-stream sample (n = 4); same number of detected isolates but different species-level composition (n = 3); and different number of isolates and their composition (n = 3). Supplementary Tables S1 and S2 show a complete list of all subjects and their EQUC findings.

A total of 18 patients had their triplets of samples subjected to both EQUC and NGS. Out of 77 EQUC-detected isolates in these 54 samples, 61 microorganisms (79%) were also detectable by NGS.

## Discussion

This is the only study to date dedicated specifically to the investigation of the potential differences among FCU, MSU and catheterised urine in men. We proved that catheterised urine samples differ in microbiota composition from spontaneously voided urine. This finding may have important consequences for future MUM studies although the margin separating the sampling methods seems somewhat narrow.

Dong et al. reported on the microbial communities of FCU samples as early as 2011^4^. The genera detected in their population of men from an STD clinic were also detected among our first-catch samples and some of them also belonged to the most abundant OTUs: *Prevotella, Streptococcus, Veillonella* and *Staphylococcus*, to name the first four. On the contrary, *Sneathia *spp. was not detected in our FCU samples at all. Different age of subjects in the Dong study (28 years), different DNA analysis method (pyrosequencing) or the variable region of the 16S rRNA gene (V1–V3) might all explain this difference but there is a substantial degree of concordance between our and Dong et al. data.

Bajic et al. were the first to note that MSU differs from catheterised samples from the same individual in their study of male lower urinary tract symptoms and suggested that catheterisation was the most appropriate method for sampling the male bladder microbiota. In a study of lower urinary tract symptoms, men with benign prostate hyperplasia were assessed for symptom severity; increase in the symptom score was associated with higher odds of detectable bacteria in catheterised urine, although no specific genera were associated with the degree of symptoms; no such association was observed when analyzing spontaneously voided urine^7^. Recently, Hourigan et al. reported significant differences in beta-diversity (but not alpha-diversity) measures between voided and catheterised urine (n = 14 men). The sample was too small to detect any particular OTU to be enriched in voided or catheterised samples, respectively^10^. Pohl et al. examined MSU and catheterised urine from 14 males of four different ethnic origins and reported differences in relative abundance of several OTUs between voided and catheterised urine^11^.

In the present study, we detected a significant difference in three out of five alpha-diversity measures—estimated number of observed genera, Chao1 and ACE indices—demonstrating that catheterised urine displays smaller degree of richness than voided urine. Beta-diversity comparisons using different stringency levels (removed singletons and doubletons, 100 most abundant OTUs among all samples and the 3 most abundant taxa per sample, respectively, Fig. 3A–C) for filtering the contingency table under the Bray–Curtis^20^ distance resulted in random patterns and no clear grouping between the three urine sampling methods. Clear patterns of microbial community dissimilarities appeared only after filtering the contingency table using the PIME^21^ method (Fig. 3D), i.e. removing noisy OTUs from the samples and keeping only the OTUs that are adding biological meaning to the group of samples (listed in Supplementary Table S3). These OTUs were not necessarily the most abundant ones, their mean relative abundance among all study samples ranging between 0.039 and 4.652% but all of them are present in 70% of samples. Some of them have not been associated with the human urinary tract so far (the genera *Ferroglobus, Streptophyta, Cutibacterium, Kiritimatiella, Rhodococcus, Candidatus Scalindua* and *Methanopyrus*). And while some of them may represent misalignment in the prokaryotic databases (such as the *Archaea, Ferroglobus* and *Methanopyrus*), others might reflect real members of the male urinary microbiota.

PIME is one of the most recent machine learning approaches to identify relevant OTUs in microbiome analyses. It has been proved useful to classify samples of patients with psychiatric disorders^22^ using only intestinal microbiota data. Such separation can be seen at the non-metric multidimensional scaling (NMDS) plot of the beta-diversity analysis. The error to prevalence trade-off is supplied as Supplementary Table S4. Clear alpha-diversity differences and the beta-diversity pattern seen in the NMDS after the PIME prevalence filtering confirm previous observations that different sections of the lower urinary tract harbour a specific microbial community. Urine obtained by catheterisation also displays a less variable community structure, with its samples more similar to each other when considering the signature OTUs identified through the Random forest models (Fig. 3D).

The topic of a “core” urinary microbiome is an intriguing one and unlike in females where *Lactobacillus, Prevotella* and *Gardnerella*^2,23^ seem to be the most commonly represented genera, there is a paucity of data on the dominant OTUs in MUM. While a study on patients from an STD clinic did not find clear evidence for a “core” microbiota in FCU samples^24^, another study employed MSU samples of 11 healthy volunteers and suggested *Streptococcus, Prevotella, Veillonella, Peptoniphilus, Campylobacter* and *Anaerococcus* to represent the core healthy MUM^25^. The only work that tried to define core MUM based on catheterised urine of males reported *Streptococcus* to be the dominant genus in males^11^. According to our data, the most common dominant genera in catheterised urine specimens include *Pseudomonas, Prevotella* and *Ferroglobus*. While the frequent presence of *Pseudomonas* and *Prevotella* in urine seems biologically plausible, *Ferroglobus spp*. is an anaerobic, Fe^2+^-oxidizing archaeum isolated from a submarine hydrothermal system^26^. There seems to be a misalignment in the process of OTU identification here which illustrates one of the weak points of current urinary microbiota investigations: public databases are inadequate for this purpose as they lack urobiome-specific genomes^27^.

Although the urinary microbiome is likely to incur some, yet undefined changes during lifetime, our data disprove the hypothesis postulated by one group that the genera *Jonquetella, Parvimonas, Proteiniphilum* and *Saccharofermentans* only dwell in bladders of people over 70 years old^28^. In our population, these OTUs were detected in 14 patients with a mean age of 66 years and some as young as 39.

The discrepancy in the yield of microorganisms from voided and catheterised urine seen in NGS extends to the study of MUM by culture. We demonstrated that voided urine of asymptomatic men with a negative standard urine culture harbours living bacteria that can be cultured using EQUC. By contrast, catheterised urine from their urinary bladders only rarely yielded a positive EQUC result. Six different sets of culture conditions were employed by our EQUC protocol. Supplementary Table S2 illustrates their respective efficacies.

Hilt et al. were the first to introduce the concept of EQUC into the study of human urinary microbiome^6^. In their landmark study, 80% of catheterised female urine specimens reported as “no growth” at 10^3^ by the standard urine culture protocol yielded a positive result using EQUC. The most prevalent genera were *Lactobacillus, Corynebacterium* and *Streptococcus*. Another study of female urinary microbiota reported 89% positive EQUC results from catheterised urine samples of 75 women not reporting signs and symptoms of UTI; most of bacterial species were not detected by standard urine culture. Median of 3 isolates per sample (IQR 1–5) were detected and *Streptococcus*, *Lactobacillus* and *Corynebacterium* (in order of prevalence) were the most prevalent genera^3^.

Several explanations can be given for the discrepancy between the yield of bacteria from catheterised urine in our and the abovementioned studies. Anatomical differences in the lower urinary tract of men and women seem the most obvious^29^: a long, twice curved male urethra as opposed to a straight, short and wide urethra in females; one that opens in direct proximity to the vaginal introit and not far from the anus. The vicinity of vaginal environment would also explain *Lactobacillus* being one of top-three bladder-dwellers in women but not in men. Another explanation for a higher detection rate of microorganisms by EQUC in the Hilt et al. and Price et al. studies^6,30^ might be less stringent enrolment criteria (no growth at 10^3^ CFU/mL and absence of UTI symptoms, respectively) compared to our study protocol. Lastly, minor modifications of our EQUC technique might have influenced the results.

In the only other work employing EQUC for the study of male urinary microbiome, Bajic et al. detected cultivable bacteria in 96% of voided urine specimens, a figure strikingly similar to our detection rate; among catheterised urine samples, 29% were positive in their study^7^. Because they did not perform standard urine culture, no comments on the pre-enrolment microbiological status of the study subjects can be made and, as noted earlier, all of our EQUC study participants preoperative urine samples were reported uniformly negative.

A subset of samples from 18 patients from our population were subjected to both EQUC and NGS in order to find out whether 16S rRNA gene sequences of the microorganisms cultured by EQUC are detectable by NGS. DNA sequences from 61 of 77 (79%) EQUC-detected microorganisms were demonstrable by NGS; for 16 isolates, NGS failed to detect their DNA. This is a recognised phenomenon reported in two other studies combining EQUC and NGS for the detection of urinary microbiota^6,8^. The culture-positive microorganisms were not necessarily the most abundant ones; on the contrary, mean relative abundance of the culture-proven microbes was 9.4% and varied widely from 0 to 93.2%.

When it comes to the limitations of the current study, the following points should be recognized. The taxonomy assignment in high-throughput sequencing amplicon studies rely on a very small segment of the marker gene, considering the whole 16S rRNA gene contains approximately 1600 base pairs; we have used fragments of around 290 bp, therefore the identification of the taxa found can be misleading, as evidenced in the case of likely misalignment of *Ferroglobus* sequences (see above). Multiple copies of the 16S rRNA marker gene can influence the abundance of the taxa identified in the study and no reliable database to correct for this bias exists so far, therefore there might be orders of magnitude errors in the ranking of the genera present in a sample. Clustering sequences into OTUs has the limitation of grouping taxa that could behave differently and multiple copies of the marker gene could separate the same taxa into two different OTUs; moreover, two OTUs belonging to the same genera could grow differently in the same treatment, being different species or even different strains of the same genus. All of these biases become noise in the identification of patterns in the microbial composition of a sample. However, these flaws are inherent to microbiota investigations in general and this study is no exception. It ought to be noted that subjects of the study were recruited from patients of a urology department and not from a healthy population. This should not be a major issue since the study focuses on intra-individual variations of the microbiota. Finally, some suggest that DNA extraction protocols that employ bead beating for cell lysis give a better representation of bacterial community structure^31^. Our method of DNA extraction relied on a commercial kit that did not employ a mechanical cell disruption procedure. It should be noted, though, that in the context of the existing microbiome literature, bead-beating is far from being standard practice.

In the present comparative study of male urinary microbiome, we corroborated previous evidence from female urinary microbiota studies regarding the detection using EQUC of living microorganisms in samples of urine that are classified as “no growth” on standard urine culture. We further demonstrated that urine samples obtained by transurethral catheterisation under aseptic conditions lead to different results on subsequent NGS analysis compared to spontaneously voided (first-catch and mid-stream) urine samples. The overall microbial community of the male lower urinary tract is too variable to see a clear difference between the three collection methods without processing and filtering of the data but there is a specific group of OTUs that can discriminate them apart. Whether the added value of such information outweighs the more invasive sampling technique will depend on the particular research question of any future male bladder microbiota study.

## Methods

This is a prospective observational study. Its objective is to investigate within-subject diversity of MUM obtained by three different methods of urine sampling and to define the most suitable approach to the study of the microbiota residing in the male urinary bladder.

### Population

The study participants were recruited among patients undergoing endoscopic procedures for benign or malignant conditions of the urinary tract in the Department of Urology, 3rd Medical Faculty of Charles University and Thomayer University Hospital, Prague, Czech Republic. To be included in the study, patients had to have a negative result of standard urine culture preoperatively, no foreign body in the urinary bladder (such as indwelling catheters, ureteric stents or bladder stones) and not have used antibiotic treatment for any medical condition in the past 6 weeks. The study was conducted in accordance with the Declaration of Helsinki after previous approval by the Ethics Committee of the Institute for Clinical and Experimental Medicine and Thomayer Hospital with Multi-center Competence under the number G-19-01 and informed consent was obtained from all participants prior to enrollment.

### Urine sampling

Participants were instructed on proper urine sample collection and were asked to provide an FCU and MSU specimens in two separate 20 mL sterile containers in the morning before surgery. A third specimen was obtained in theatre at the beginning of the procedure after disinfection of the genital area, surgical draping and immediately upon endoscope insertion. A water-based jelly not containing chlorhexidine or any other desinfectant (Optilube, Optimum Medical Solutions, Leeds, UK) was used to minimize the chances of harm to any presumed living intravesical microorganisms. All samples were stored at 4 °C before inoculation and processed on the same day with EQUC or frozen and stored at − 20 °C until DNA extraction.

### Expanded quantitative urine culture

Only subjects with “no growth” reported on routine preoperative culture were eligible for the EQUC part of the study. Patients with preoperative cultures deemed negative for clinical purposes but reported as “commensal flora” or “suspected contamination” were excluded. Antibiotic prophylaxis, where indicated, was administered after urine samples were obtained. EQUC protocol was based on previous description by Hilt et al^6^. A 100 µL aliquot of each urine sample was inoculated with a sterile plastic loop onto agar plates (90 mm in diameter) and in liquid broth. EQUC culture conditions were as follows: (1) Columbia blood agar (CBA) incubated at 37 °C for 48 h; (2) CBA incubated at 30 °C for 48 h; (3) CBA incubated at 37 °C in a 5% CO_2_ incubator for 48 h; (4) Chocolate agar incubated at 37 °C in a 5% CO_2_ incubator for 48 h; (5, 6) Schaedler blood agar at 37 °C in a Campy gas mixture (5% O_2_, 10% CO_2_ and 85% N) in Oxoid Anaerojar; (7) thioglycolate broth incubated at 37 °C for 5 days, then inoculated on CBA and incubated at 37 °C for another 48 h. CBA and Schaedler agar were prepared in the laboratory from dried base (Bio-Rad, Berkeley, CA, USA). 5% sheep blood (LMS—Labmediaservis, Czech Republic) was added into the medium at 38 °C. Thioglycolate broth was prepared from dehydrated powder (Bio-Rad, Berkeley, CA, USA). Chocolate agar was obtained as commercial product (Bio-Rad, Berkeley, CA, USA).

The number of colonies was then counted on agar plates with inoculated urine. Growth was visually detected in the thioglycolate broth and colonies were counted on CBA after inoculation and incubation.

Matrix assisted laser desorption ionization–time of flight mass spectrometry (MALDI-TOF, Bruker Daltonik GmbH, Leipzig, Germany) was used for the identification of individual bacteria.

### DNA extraction and PCR

Bacterial 16S rRNA gene was extracted from urine samples using Eligene Urine Isolation Kit (Elisabeth Pharmacon, Brno, Czech Republic) according to manufacturer’s instructions. Briefly, the whole amount of urine was vortexed for 15 s, 10 mL of urine was then centrifuged at 6000×*g* for 20 min, the supernatant was discarded and pellet resuspended in 200 μL of molecular grade water, 200 μL of MI3 solution, and 20 μL of Proteinase K was added. After 15 s vortexing, the mixture was incubated for 15 min at 65 °C. The lysate was cetrifuged at 6000×*g* for 5 min. The supernatant was transferred to microtube and 210 μL of MI4 solution added. The lysate was centrifuged for 1 min at 13,000×*g*. DNA extraction controls as well as negative controls for PCR reactions were included.

The primers 515F (5-GTGCCAGCMGCCGCGGTAA) and 806R (5-GGACTACHVGGGTWTCTAAT) were used to amplify the hypervariable region V4 of the 16S rRNA gene. Each forward primer was barcoded by a sequence nucleotides designed to multiplexing of different samples^32^. PCR was performed in triplicates on each sample, and every reaction contained 5 μL of 5xQ5 Reaction Buffer for Q5 High-Fidelity DNA polymerase; 0.25 μL Q5 High-Fidelity DNA polymerase; 5 μL of 5xQ5 HighGC Enhancer; 1.5 μL of BSA (10 mg/mL); 0.5 μL of PCR Nucleotide Mix (10 mM); 1 μL of primer 515F (10 μM); 1 μL of primer 806R (10 μM,); 1.0 μL of template DNA and sterile ddH2O up to 25 μL. Conditions for amplification started at 94 °C for 4 min followed by 25 cycles of 94 °C for 45 s, 50 °C for 60 s, 72 °C for 75 s and finished with a final setting of 72 °C for 10 min. The triplicates were pooled together, purified with the MinElute PCR Purification Kit (Qiagen) to remove the enzymes and compounds required for the PCR reaction, and mixed in equimolar amounts according to the concentration measured with the Qubit 2.0 Fluorometer (Thermo Fisher Scientific). The sequencing library was prepared using the TruSeq DNA PCR-Free LP Kit (Illumina) by ligation of adaters TruSeq DNA SgI Index according to the manufacturer’s instructions. The ligated library was quantified using KAPA Library Quant Kit (Roche) and pooled with libraries from other sequencing projects to obtain final library (30 μL with a concentration of 4 nM, confirmed by KAPA Library Quant Kit). PhiX Control (Illumina) with a total of 2% of the run was used as a spike-in control. Sequencing was performed on Illumina MiSeq (2 × 250 bases) using the MiSeq v2 Reagent Kit (Illumina).

### Statistical analyses

Demographic and clinical data were analysed as continuous or categorical variables and reported as median and inter-quartile range (IQR) or percentages as appropriate. Bacterial growth using EQUC was assessed as present or absent and correlation between the three samples from individual patients were evaluated. XLSTAT (Addinsoft, New York, USA) was used for statistical calculations.

The sequencing data were processed using SEED 2.1.2^33^. Pair-end reads were merged using fastq-join^34^. Sequences with ambiguous bases were omitted as well as sequences with average quality PHRED score < 30, those shorter than 280 bases and longer than 320 bases. The chimeric sequences were detected and removed using Usearch 7.0.1090, and clustered into OTUs using the uparse agorithm^35^ at a 97% similarity level. The most abundant sequence from each cluster^36^ was assigned to the closest hits at the genus level using the RDP Naïve Bayesian Classifier Version 2.11 method^37^ for bacteria. Sequences identified as non-bacterial were discarded. The DNA sequences have been deposited at the NCBI SRA under the accession number PRJNA744742. Analyses of alpha- and beta-diversity were perfomed using the packages Vegan^38^, phyloseq^39^ and Prevalence Interval for Microbiome Evaluation (PIME)^21^ from R language^40^. The PIME algorithm is used to identify the most relevant OTUs to separate treatments or groups of samples. The method is based on the concept of prevalence, considering that high abundance OTUs that have low prevalence among samples from the same treatment/group are not relevant to characterise a treatment/group of samples. It uses Random Forests to classify the groups of samples using high prevalence OTUs. For alpha-diversity analyses, the data were normally distributed and block design ANOVA was employed; alpha-level of 0.05 was considered statistically significant.

## Supplementary Information

Supplementary Table S1.

Supplementary Table S2.

Supplementary Table S3.

Supplementary Table S4.

Supplementary Table S5.

## Data Availability

The raw data generated and/or analysed during this study are included in this published article as supplementary files. Larger datasets (16S rrNA gene sequences) are available in the NCBI SRA database under the accession number PRJNA744742 (http://www.ncbi.nlm.nih.gov/bioproject/744742). Supplementary table S5 contains information on the collection approach for each sample.
