# Supplementary material for: Alpha-diversity and microbial community structure of the male urinary microbiota depend on urine sampling method
Source: Sci Rep. 2021 Dec 9;11:23758. doi: 10.1038/s41598-021-03292-x (PMC8660768; doi:10.1038/s41598-021-03292-x)
Supplement: Supplementary file 2 — Supplementary Table S2. [file 41598_2021_3292_MOESM2_ESM.pdf]

Sample type (A, B, C)	Microorganism isolated	BAP CO_2	Chocolate agar CO_2	BAP 35 ˚C	BAP 30 ˚C	Schaedler agar	Schaedler agar	Thioglycolate broth
A	Staphylococcus haemolyticus	≥100	≥100	≥100	≥100	≥100	≥100	Staphylococcus haemolyticus
B	Enterococcus faecalis	49	47	41	30	34	35	Enterococcus faecalis
C		0	0	0	0	0	0	
A	Corynebacterium glucuronolyticum	5	7	9	2	2	4	
	Staphylococcus lugdunensis	0	0	3	2	2	0	
	Streptococcus mitis	21	40	30	4	18	24	
B	Corynebacterium glucuronolyticum	60	5	69	45	0	5	Corynebacterium glucuronolyticum
	Staphylococcus lugdunensis	8	5	7	5	4	3	Staphylococcus lugdunensis
	Streptococcus mitis	18	24	21	14	16	19	Streptococcus mitis
								Staphylococcus epidermidis
C		0	0	0	0	0	0	
A	Actinotignum schaalii	0	0	0	0	2	5	
		0	0	0	0	0	0	
B		0	0	0	0	0	0	
C		0	0	0	0	0	0	
A	Corynebacterium glucuronolyticum	≥100	21	≥100	≥100	≥100	≥100	Corynebacterium glucuronolyticum
	Enterococcus faecalis	23	0	16	0	22	21	Enterococcus faecalis
B	Corynebacterium glucuronolyticum	84	1	56	≥100	56	63	Corynebacterium glucuronolyticum
	Enterococcus faecalis	7	5	4	7	8	6	Enterococcus faecalis
C		0	0	0	0	0	0	
A	Staphylococcus epidermidis	0	0	0	20	0	0	Staphylococcus epidermidis
B		0	0	0	0	0	0	
C		0	0	0	0	0	0	
A	Staphylococcus epidermidis	60	0	5	≥100	5	5	Staphylococcus epidermidis
	Actinotignum schaalii	0	0	18	0	2	7	
	Enterococcus faecalis	0	0	0	0	4	3	
B	Staphylococcus epidermidis	9	2	3	7	2	5	Staphylococcus epidermidis
	Actinotignum schaalii	0	1	6	20	3	3	
	Enterococcus faecalis	0	0	0	0	2	5	
C		0	0	0	0	0	0	
A	Streptococcus mitis	48	19	11	0	18	7	Streptococcus mitis
	Moraxella osloensis	9	12	18	38	4	3	Moraxella osloensis
	Actinomyces urogenitalis	9	1	1	0	0	0	Actinomyces urogenitalis
B	Streptococcus mitis	23	20	16	15	14	28	Streptococcus mitis
	Streptococci	≥100	6	≥100	0	24	32	Streptococci
	Actinomyces urogenitalis	43	38	25	3	5	4	Actinomyces urogenitalis
C		0	0	0	0	0	0	
A	Staphylococcus epidermidis	9	21	6	29	9	8	Staphylococcus epidermidis
	Staphylococcus haemolyticus	0	0	0	0	0	2	Staphylococcus haemolyticus
	Enterococcus faecalis	0	0	0	0	0	1	Enterococcus faecalis
B	Staphylococcus epidermidis	7	18	7	7	4	9	Staphylococcus haemolyticus
	Staphylococcus haemolyticus	56	0	≥100	27	5	2	Enterococcus faecalis
	Enterococcus faecalis	4	4	2	4	3	48	
C		0	0	0	0	0	0	
A	Enterococcus faecalis	19	32	21	0	0	2	Enterococcus faecalis
	Staphylococcus epidermidis	0	0	0	≥100	0	27	Staphylococcus epidermidis
B	Enterococcus faecalis	58	47	56	0	48	45	Enterococcus faecalis
	Staphylococcus epidermidis	0	0	0	0	2	2	Staphylococcus epidermidis
C		0	0	0	0	0	0	
A	Streptococcus mitis	76	≥100	68	89	≥100	≥100	Streptococcus mitis
	Staphylococcus haemolyticus	25	4	7	2	3	5	Staphylococcus haemolyticus
B	Streptococcus mitis	7	9	8	7	4	6	Streptococcus mitis
	Staphylococcus epidermidis	2	1	2	2	2	3	Staphylococcus epidermidis
C		0	0	0	0	0	0	
A	Streptococcus salivarious	15	13	25	12	27	16	Streptococcus salivarious
	Staphylococcus epidermidis	6	3	3	9	1	4	Staphylococcus epidermidis
	Enterococcus faecalis	32	42	0	0	0	0	Enterococcus faecalis
B	Streptococcus salivarius	9	4	18	7	10	6	Streptococcus salivarius
	Staphylococcus epidermidis	16	24	8	5	5	7	Staphylococcus epidermidis
	Enterococcus faecalis	16	24	0	0	0	0	Enterococcus faecalis
C		0	0	0	0	0	0	
A	Enterococcus faecalis	42	6	35	6	8	10	Enterococcus faecalis
	Staphylococcus epidermidis	27	16	21	26	16	30	Staphylococcus epidermidis
B	Enterococcus faecalis	6	1	4	0	0	0	Enterococcus faecalis
	Staphylococcus epidermidis	4	3	4	3	4	2	Staphylococcus epidermidis
C	Enterococci	0	4	2	0	0	0	Enterococci
	Staphylococcus epidermidis	0	2	2	0	0	0	Staphylococcus epidermidis
A	Streptococcus mitis	24	15	18	12	0	0	Streptococcus mitis
	Staphylococcus saprophyticus	10	0	8	24	12	28	Staphylococcus saprophyticus
	Enterococcus faecalis	18	0	21	20	≥100	≥100	Enterococcus faecalis
B	Streptococcus mitis	7	3	6	8	0	0	Streptococcus mitis
	Staphylococcus saprophyticus	8	0	16	5	6	10	Staphylococcus saprophyticus
	Enterococcus faecalis	36	2	33	18	≥100	≥100	Enterococcus faecalis

C		0	0	0	0	0	0	
A	Staphylococcus epidermidis	≥100	87	96	65	≥100	≥100	Staphylococcus epidermidis
B	Staphylococcus epidermidis	40	41	33	18	46	31	Staphylococcus epidermidis
C		0	0	0	0	0	0	
A	Enterococcus faecalis	6	5	4	3	8	9	Enterococcus faecalis
	Staphylococcus epidermidis	1	1	1	0	8	3	Staphylococcus epidermidis
B		0	0	0	0	0	0	
C		0	0	0	0	0	0	
A	Enterococcus avium	7	3	4	2	8	4	Enterococcus avium
	Corynebacterium glucuronicum	4	0	1	0	15	12	Corynebacterium glucuronicum
B		0	0	0	0	0	0	
C		0	0	0	0	0	0	
A	Corynebacterium glucuronicum	57	75	71	21	83	10	Corynebacterium glucuronicum
	Enterococcus faecalis	26	19	4	15	6	30	Enterococcus faecalis
B	Corynebacterium glucuronicum	48	26	44	32	43	0	Corynebacterium glucuronicum
	Enterococcus faecalis	22	26	12	14	12	2	Enterococcus faecalis
C		0	0	0	0	0	0	
A	Streptococcus agalactiae	34	32	19	26	16	10	Streptococcus agalactiae
	Streptococcus anginosus	13	8	14	11	6	30	Streptococcus anginosus
B	Streptococcus agalactiae	9	20	9	7	17	0	Streptococcus agalactiae
	Streptococcus anginosus	8	13	9	3	12	2	Streptococcus anginosus
C		0	0	0	0	0	0	
A	Streptococcus anginosus	47	32	59	0	contamination	contamination	Streptococcus anginosus
	Corynebacterium glucuronicum	7	0	3	6	contamination	contamination	Corynebacterium glucuronicum
	Alloscardovia omnicolens	0	20	0	0	contamination	contamination	Alloscardovia omnicolens
B	Streptococcus anginosus	29	13	15	0	contamination	contamination	Streptococcus anginosus
	Corynebacterium glucuronicum	17	5	29	4	contamination	contamination	Corynebacterium glucuronicum
	Alloscardovia omnicolens	0	2	0	0	contamination	contamination	Alloscardovia omnicolens
C		0	0	0	0	contamination	contamination	
A	Streptococcus anginosus	≥100	87	≥100	≥100	60	55	Streptococcus anginosus
	Streptococcus mitis	≥100	38	≥100	9	27	20	Streptococcus mitis
	Corynebacterium glucuronicum	21	16	20	4	9	11	Corynebacterium glucuronicum
B	Streptococcus anginosus	≥100	28	≥100	15	62	76	Streptococcus anginosus
	Streptococcus mitis	≥100	15	15	0	38	33	Streptococcus mitis
	Corynebacterium glucuronicum	11	6	6	0	15	16	Corynebacterium glucuronicum
C	Corynebacterium glucuronicum	18	0	0	0			Corynebacterium glucuronicum
A	Staphylococcus hominis	67	36	26	37	41	52	Staphylococcus hominis
	Staphylococcus lugdunensis	12	8	12	13	11	14	Staphylococcus lugdunensis
B	Staphylococcus hominis	56	48	39	45	52	56	Staphylococcus hominis
	Staphylococcus lugdunensis	14	32	28	29	23	22	Staphylococcus lugdunensis
C		0	0	0	0	0	0	
A	Corynebacterium glucuronicum	46	7	≥100	≥100	14	17	Corynebacterium glucuronicum
	Actinotignum schaalii	4	3	10	2	0	0	Actinotignum schaalii
B	Corynebaterium glucuronicum	51	1	≥100	≥100	8	9	Corynebaterium glucuronicum
	Staphylococcus hominis	6	1	3	3	1	2	Staphylococcus hominis
C		0	0	0	0	0	0	
A		0	0	0	0	0	0	
B	Lactobacillus curvatus	11	0	3	12	5	3	
	Staphylococcus hominis	2	3	0	2	1	0	
C		0	0	0	0	0	0	
A	Veilonella criceti	6	30	4	6	21	18	
	Enterococcus fecalis	8	10	13	13	2	1	Enterococcus fecalis
B	Veilonella criceti	1	0	2	0	0	0	
	Enterococcus fecalis	1	0	1	0	2	0	
C	Enterococcus fecalis	1	0	1	0	0	2	Enterococcus fecalis
A	E. coli	≥100	≥100	≥100	≥100	≥100	≥100	E. coli
B	E. coli	≥100	56	≥100	72	78	63	E. coli
C		0	0	0	0	0	0	
A	Streptococcus oralis	82	38	70	32	28	20	Streptococcus oralis
	Stahlylococcus sp., NS	5	2	6	2	2	2	
B	Streptococcus oralis	≥100	≥100	≥100	51	15	12	Streptococcus oralis
	Stahlylococcus sp., NS	7		6	4	3	2	Stahlylococcus sp., NS
C		0	0	0	0	0	0	
A	Stapnylococcus equorum	6	0	5	4	4	5	Stapnylococcus equorum
	Stapnylococcus epidermidis	35	26	28	33	30	32	Stapnylococcus epidermidis
B	Stapnylococcus equorum	6	1	11	7	0	1	Stapnylococcus equorum
	Stapnylococcus epidermidis	10	8	3	4	6	5	Stapnylococcus epidermidis
C		0	0	0	0	0	0	
A	Dermabacter hominis	4	2	0	0	1	0	Dermabacter hominis
B	Dermabacter hominis	2	2	1	0	0	0	Dermabacter hominis
C		0	0	0	0	0	0	
A	Stapnylococcus epidermidis	5	5	8	1	1	0	Stapnylococcus epidermidis
	Streptococcus oralis	6	2	6	2	2	2	
B	Stapnylococcus epidermidis	3	2	3	0	1	0	Stapnylococcus epidermidis
	Streptococcus oralis	1	2	1	0	0	0	

C		0	0	0	0	0	0	
A	Staphylococcus hominis	5	19	12	6	12	8	Staphylococcus hominis
	Corynebaterium glucuronicum	21	56	29	15	74	80	
	Streptococcus anginosus	48	≥100	≥100	43	≥100	≥100	Streptococcus anginosus
B	Staphylococcus hominis	2	1	1	0	6	5	Staphylococcus hominis
	Corynebaterium glucuronicum	14	18	12	7	20	13	
	Streptococcus anginosus	31	57	46	28	43	33	Streptococcus anginosus
C		0	0	0	0	0	0	
A	Brevibacterium paucivorans	0	0	3	0	1	0	Brevibacterium paucivorans
	viridans streptococci, NS	1	0	1	0	1	1	viridans streptococci, NS
B	Brevibacterium paucivorans	5	7	6	2	0	0	Brevibacterium paucivorans
	viridans streptococci, NS	1	2	2	2	0	0	viridans streptococci, NS
C		0	0	0	0	0	0	

Supplementary table S2: Detection performance (numbers of colonies) of individual culture conditions in the EQUC protocol.
A: first catch urine, B: mid-stream urine, C: catheterised urine, NS: not specified
